# Microbicidal Activity of Hypothiocyanite against Pneumococcus

**DOI:** 10.3390/antibiotics10111313

**Published:** 2021-10-28

**Authors:** Edriss Yassine, Balázs Rada

**Affiliations:** Department of Infectious Diseases, College of Veterinary Medicine, University of Georgia, 501 D.W. Brooks Dr., Athens, GA 30602, USA; edriss.yassine25@uga.edu

**Keywords:** *Streptococcus pneumoniae*, hypothiocyanite, microbicidal, pneumococcus, OSCN-

## Abstract

Infections caused by *Streptococcus pneumoniae* (pneumococcus, *Spn*) manifest in several forms such as pneumonia, meningitis, sinusitis or otitis media and are associated with severe morbidity and mortality worldwide. While current vaccines and antibiotics are available to treat *Spn* infections, the rise of antibiotic resistance and limitations of the vaccines to only certain *Spn* serotypes urge the development of novel treatments against *Spn*. Hypothiocyanite (OSCN-) is a natural antimicrobial product produced by the body’s own innate immune system to fight a variety of pathogens. We recently showed that OSCN- is also capable of killing *Spn* in vitro. OSCN- is an oxidative agent attacking microbes in a nonspecific manner, is safe for the host and also has anti-inflammatory effects that make it an ideal candidate to treat a variety of infections in humans. However, OSCN- has a short life span that makes its use, dosage and administration more problematic. This minireview discusses the antimicrobial mechanism of action of OSCN- against *Spn* and elaborates on the potential therapeutic use of OSCN- against *Spn* and other infectious agents, either alone or in combination with other therapeutic approaches.

## 1. Introduction

*Streptococcus pneumoniae* (*Spn*, pneumococcus) is a gram-positive, opportunistic bacterium that can be found as part of the normal human flora in the upper respiratory tract of children and adults [1]. Pneumococcal disease can manifest in many forms such as pneumonia, meningitis, sinusitis or otitis media [2]. The World Health Organization (WHO) estimates that infections with *Spn* kill more than 500,000 children under 5 years of age each year [2,3], and *Spn* is responsible for over 150,000 annual hospitalizations in the United States alone. There are currently two approved pneumococcal vaccines in use that work by targeting the capsular polysaccharide of that bacterium, PCV13 and PPSV23, the number at the end representing the number of serotypes covered by the vaccine. Despite their availability, a limitation of the vaccines is that they are serotype-specific. Currently, there are 100 identified serotypes of *Spn* [4] and the vaccines only provide protection against less than 25% of the circulating serotypes. *Spn* infections can be treated with the use of antibiotics, however, like many other diseases treated in this manner, the rise of antibiotic resistance is becoming a critical problem and there is a need for novel therapeutics [5]. 

The epithelial layer of the airway passages constitutes the first line of defense, both physical and immunological, against invaders that are inhaled in the body [6,7]. One of the antimicrobial substances used by the airway epithelium is the antimicrobial oxidizing agent, hypothiocyanite (OSCN-). In the airways, thiocyanate (SCN-) is oxidized by H_2_O_2_ in the presence of lactoperoxidase (LPO) to form OSCN- [8,9]. This system has been shown to have in vitro microbicidal activity against a wide variety of pathogens including bacteria [9,10,11,12], viruses [13,14,15,16] and fungi [17]. A comprehensive table of such organisms can be found in our review [12]. Due to the fact that components of this system can be naturally found in the body and OSCN- is a powerful microbicidal agent, the potential of using it as an alternative therapeutic agent should be explored further.

## 2. Reactive Oxygen Species (ROS)

ROS represent one of the first lines of defense of the innate immune system. When pathogens are recognized, one of the first innate immune responses aiming at their eradication is the production of ROS by different cells in the body, including airway epithelial cells, neutrophils and macrophages [18,19]. OSCN- is one form of ROS that can be found in many bodily secretions including the saliva and the airway surface liquid [20,21] where it acts as an antimicrobial agent against a variety of microbes including bacteria [22,23]. NADPH oxidases are among the main sources of endogenous ROS in the body. In the airways, dual oxidases 1 and 2 (DUOX1 and DUOX 2) are major sources of H_2_O_2_ that is used to convert SCN- to OSCN- in the presence of LPO [8,9]. This family of transmembrane proteins transfer an electron from NADPH to an FAD cofactor followed by a transfer to a heme group which generates the final oxidative product [24,25,26]. Lactoperoxidase (LPO) belongs to the family of heme peroxidases whose primary role is to act as oxidoreductase enzymes [27,28]. In the airway epithelium, LPO is secreted by submucosal glands and epithelial cells [29,30]. SCN- is a pseudohalide that is also found in abundance in the airway surface liquid [31,32]. LPO uses H_2_O_2_ to oxidize SCN- to form OSCN-. The formation of OSCN- has multiple benefiting factors to the host as it targets a broad range of microorganisms, and it also acts as an anti-inflammatory substance by scavenging neutrophil-generated hypochlorous acid, the main cause of neutrophil-mediated oxidative tissue damage. The wide spectrum of microbes targeted by OSCN- represents an advantage over antibiotics since antibiotics have a narrower target range. This is due to the fact that antibiotics have specific targets (i.e., the cell wall, nucleic acid synthesis or inhibition of metabolic pathways) [33]. Contrary to these traditional targets, OSCN- has the ability to oxidatively target a wide range of species, including bacteria [9,10,11,12], viruses [13,14,15,16] and fungi [17], due to its nonspecific nature using ROS. However, one major drawback of OSCN- is its short half-life [34], making it a difficult substance to work with, both in a laboratory setting as well as in vivo. 

## 3. Hypothiocyanite 

OSCN- has a few described mechanisms by which it kills microorganisms. For example, in certain bacteria, including *E. coli*, OSCN- gets into the bacterium by using porins and hydrophobic channels, and once inside, it has the ability to block the uptake of essential molecules, like glucose, potassium and amino acids and causes the leakage of these substances from the bacterial cells themselves by interfering with the transport proteins needed for the intake of these substances [35,36]. Furthermore, in *Streptococcus* sp., OSCN- has the ability to hinder the bacterium’s metabolism by targeting glycolytic enzymes and oxidizing glucose uptake transporters, ultimately leading to cell death [37,38]. Lastly, the simple presence and abundance of OSCN- in the oral cavity has been shown to inhibit acid production and reduce the number of bacteria that can cause dental plaques [20]. The effects of OSCN- can be demonstrated in other manners, as well. For example, we showed that OSCN- has the ability to inactivate several strains of influenza A and B viruses in vitro using a cell-free approach [15] and that similar viral inactivation can be observed on the apical surface of primary tracheobronchial epithelial cells derived from rats and humans [14]. Furthermore, we also determined that OSCN- does not inactivate the virus by priming host cells but by directly inactivating the virus when OSCN- is generated at the time of viral replication [39]. Lastly, our study showed that viral RNA synthesis in host cells was also inhibited by OSCN-, most likely as a downstream event while no physical damage to the viral structure itself was observed [39]. Recently, there have been some concerns raised about the toxicity of OSCN-, more specifically its protonated form, hypothiocyanous acid (HOSCN) [40]. Although, as mentioned previously, OSCN- is nontoxic to cells in the oral cavity and the airway epithelium, some studies have suggested that HOSCN can induce cellular damage and cytotoxicity to certain cell types including red blood cells [41] and endothelial cells located in the arteries [42]. These aspects still need to be studied further. 

## 4. OSCN- and *S. pneumoniae*

Data describing the general antibacterial effects of OSCN- have been accumulated for several decades by now. OSCN- has been shown to be effective against several *Streptococcus* sp. in vitro [12]; however, we were the first laboratory to show that it is also effective against *Spn*. In our recent work, we studied the effects of OSCN- on different serotypes of *Spn*, specifically those that contain or lack a protective capsule [9]. Using a cell-free in vitro system containing LPO/SCN-/H_2_O_2_, we were able to show that SCN- was converted to OSCN- and there was effective killing of both encapsulated and non-encapsulated variants of *Spn*. To verify that the H_2_O_2_ present in the system was indeed converting SCN- to OSCN-, we showed that the addition of catalase into a cell-free system rescued the growth of *Spn* by scavenging H_2_O_2_. To further test the effects of OSCN- on *Spn* growth, we showed that *Spn* growth was also inhibited when a commercially available product, 1st line, was used that generates OSCN- and allows it to be stable for longer periods of time [9]. The mechanism of action of OSCN- against *Spn* is still not fully understood; however, we were able to show that OSCN- does not initiate *Spn* killing via the *lytA*-mediated autolytic pathway since wild-type and *lytA*-deficient strains of *Spn* were both killed by OSCN- [9]. Since *Spn* is also able to produce H_2_O_2_ on its own via the pyruvate oxidase pathway, we wanted to determine if this endogenous H_2_O_2_ could possibly interfere with OSCN- killing of *Spn*. By using wild-type bacteria and an isogenic strain deficient in the pyruvate oxidase gene, we determined a partial but significant decrease in the sensitivity of the pyruvate oxidase-deficient strain to OSCN- compared to the control cells. Thus, pyruvate oxidase does provide some limited protection for *Spn* against OSCN-, possibly by boosting bacterial oxidant defenses [9]. However, the exact mechanism of action of this observation remains to be determined in the future.

## 5. OSCN- as a Therapeutic

There is a therapeutic currently being tested in cystic fibrosis patients that contains OSCN-, ALX-109. ALX-109 is a combination of lactoferrin and OSCN-, both naturally occurring substances in the human innate immune system [10]. Lactoferrin is an iron-binding glycoprotein that is commonly found in mammalian secretions and has the ability to prevent bacterial growth by either sequestering iron or by generating hydroxyl radicals [43]. When combined with OSCN-, the interaction becomes synergistic. When ALX-109 was inhaled, in combination with antibiotics such as tobramycin or aztreonam, it was able to reduce biofilm formation of *Pseudomonas aeruginosa* as well as remain nontoxic to the cells of the upper airways over long periods of time [10]. In a separate study, Tunney et al. showed that ALX-109 was able to decrease the levels of four different strains of *P. aeruginosa* to below detectable levels in 2 h and significantly decrease pathogen levels in sputum samples of cystic fibrosis patients within 6 h of incubation [34]. The combination of lactoferrin and OSCN- not only has bactericidal effects, but was also shown to be fungicidal. Nakano et al. showed the effectiveness of this combination against *Candida albicans*, a common opportunistic fungus that can colonize humans [17]. Furthermore, we also showed that the drug 1st line also has bactericidal and virucidal effects in vitro [9,39]. These examples propose that OSCN- does have the potential to be used as a therapeutic against common pathogens including *Spn*. Although promising, the use of OSCN- in humans as a potential anti-*Spn* therapeutic is still very much unexplored. An SCN- analog, selenocyanate (SeCN^−^), was also tested against respiratory cystic fibrosis bacterial pathogens in vitro and was found to be more effective in bacterial killing than OSCN- itself [44]. Thus, not only SCN-, but also SeCN^−^ should be explored for pneumococcal killing. While the individual components have been deemed safe due to the fact that they are naturally occurring in the human body, it is still yet to be determined if these components can be delivered in a manner that will be conducive of a therapeutic drug. ALX-109 is inhaled and seems to be working in clinical tests; however, it remains to be determined whether a combination with lactoferrin is the most impactful therapeutic use of OSCN-. There is also the question whether OSCN- or the components that generate it can be given orally? To be effective, the protein components would need to evade degradation in the stomach, be absorbed in the blood and find their way to the airways. The short life span of OSCN- represents a considerable challenge for its therapeutic use since all the enzymatic components of the OSCN- generating system need to be applied, potentially directly at the site of their intended target. New techniques either increasing the stability of OSCN- or providing alternative mechanisms of OSCN- generation/release would likely improve its therapeutic efficacy. Studies also need to investigate whether using SCN- alone, the prodrug of OSCN-, is sufficient and the therapy can rely on the body’s own H_2_O_2_ generation and LPO activity. It is also yet to be determined if OSCN- would be best taken as a prophylactic to prevent disease or as a therapeutic after the establishment of disease caused by *Spn* and other infectious agents.

## 6. Concluding Remarks

OSCN- itself, as well as its counterparts LPO and SCN-, are all naturally occurring substances in the body and have the potential to be safe for use as therapeutics to treat a wide array of infections in humans. Studies have shown that OSCN- is effective at having microbicidal activity against many different species of organisms including bacteria such as *Spn*, viruses and fungi with minimal or no consequences to the host. This is promising as the cases of antibiotic resistance have been on the rise and many species including *Spn* have become multidrug-resistant. The ability of OSCN- to work in a different manner than traditional antibiotics or vaccines brings hope that in the future there may be a way to implement it as a single or combination therapy to circumvent the problem of drug resistance. It is promising that there are already therapeutics in the testing phase which contain OSCN-; however, more studies are needed to determine the most effective and safe manner of OSCN- delivery in humans.

## Abbreviations

ROS: reactive oxygen species; OSCN-, hypothiocyanite; Spn, *Streptococcus pneumoniae*; H_2_O_2_, hydrogen peroxide; LPO, lactoperoxidase; SCN-, thiocyanate; Duox, dual oxidase.
